# Supplementary material for: COVID-19 vaccination acceptance among dental students and dental practitioners: A systematic review and meta-analysis
Source: PLoS One. 2022 Apr 19;17(4):e0267354. doi: 10.1371/journal.pone.0267354 (PMC9017896; doi:10.1371/journal.pone.0267354)
Supplement: S1 Table — (PDF) [file pone.0267354.s001.pdf]

S1 Table. Subgroup analyses of geographical regions and country income levels on the acceptance rates (%) of COVID-19 vaccine among dental practitioners.

**Geographical regions**								
**Middle East**			**Europe**			**South Asia**		
Weighted mean (%)	Confidence intervals		Weighted mean (%)	Confidence intervals		Weighted mean (%)	Confidence intervals	
	Upper bound	Lower bound		Upper bound	Lower bound		Upper bound	Lower bound
85.6	91.5	75.8	82.2	85.6	78.9	72.6	75.8	71.4

**Country income levels**								
**Lower-middle**			**Upper-middle**			**High**		
Weighted mean (%)	Confidence intervals		Weighted mean (%)	Confidence intervals		Weighted mean (%)	Confidence intervals	
	Upper bound	Lower bound		Upper bound	Lower bound		Upper bound	Lower bound
72.6	75.8	71.4	84.0	89.3	78.7	86.0	89.0	83.1
