# Supplementary material for: COVID-19 vaccination acceptance among dental students and dental practitioners: A systematic review and meta-analysis
Source: PLoS One. 2022 Apr 19;17(4):e0267354. doi: 10.1371/journal.pone.0267354 (PMC9017896; doi:10.1371/journal.pone.0267354)
Supplement: S2 Table — (DOCX) [file pone.0267354.s002.docx]

**S2 Table.** Meta-regression evaluating the effect of sample size of each study on the acceptance rates (%) of COVID-19 vaccine among dental students and dental practitioners.

|  | Coefficient | Confidence intervals | | Standard error | *P*-value |
| --- | --- | --- | --- | --- | --- |
|  |  | Upper bound | Lower bound |  |  |
| Dental students | 0.577 | 0.617 | 0.537 | 0.021 | 0.060 |
| Dental practitioners | 0.857 | 1.059 | 0.655 | 0.103 | 0.611 |
