# Supplementary material for: COVID-19 vaccination acceptance among dental students and dental practitioners: A systematic review and meta-analysis
Source: PLoS One. 2022 Apr 19;17(4):e0267354. doi: 10.1371/journal.pone.0267354 (PMC9017896; doi:10.1371/journal.pone.0267354)
Supplement: Attachment — Submitted filename: Response to Reviewers.docx [file pone.0267354.s005.docx]

**Response to Reviewers**

The authors would like to thank the editor and reviewers for considering the manuscript and their valuable comments. The amendments are made in the revised manuscript.

| **No.** | **Reviewer comment** | **correction** |
| --- | --- | --- |
| Reviewer 1 | | |
| 1. | Add more information in the background section, such as the purpose of study, etc. | The background has been amended.  Page 2, Line 3-6:  ‘*The contemporary COVID-19 pandemic has prompted researchers across the world …… acceptance towards COVID-19 vaccination is still scarce.*’ |
| 2. | Introduction: Minor information on the variants of COVID-19 and their future challenges can be included i.e., Doi: 10.1007/s15010-021-01734-2. | Several points have been added to the introduction.  Page 4:  “*Several strains of SARS-CoV-2 have been discovered throughout the pandemic and are categorized into three groups …… to curb the widespread of the viruses*” |
| 3. | The English may be improved (Minor). | The manuscript has been proofread by an English native speaker. |
| 4. | The graphical figure resolution is low. It has to be improved for publication. It isn't easy to interpret results from them. | The authors would like to thank the reviewer for raising this concern. High-resolution figures (Figure 1 and Figure 2) will be provided to the journal editor. |
| 5. | Proper figure legends are also missing from the manuscript. | Figure legends were written in the text.  Page 9:  “*Fig 1. PRISMA flowchart. Study selection …… PRISMA guidelines*”  Page 13  “*Fig 2. Meta-analysis of COVID-19 vaccine acceptance …… and dental practitioners*.” |
| 6. | The authors should cross-check all abbreviations in the manuscript. Provide all abbreviations in a separate paragraph. | All abbreviations have been cross-checked and a separated section ‘Abbreviation’ was added into the manuscript. |
| 7. | Add the limitation of the manuscript in a separate section. | A separated section of limitation has been added. |
| Reviewer 2 | | |
| 1. | Introduction, please include some quantitative information about COVID-19 variants and their challenges in its prevention. | Several points have been added to the introduction.  Page 4:  “*Several strains of SARS-CoV-2 have been discovered throughout the pandemic and are categorized into three groups …… to curb the widespread of the viruses*” |
| 2. | Discussion, please highlight minor information about cases, mortality, and casualty of COVID-19 in case of dental students and dental practitioners. | The authors added few points in the discussion.  Page 16:  “*Despite the significant likelihood of being infected by the viruses, …… the increased vaccine acceptability and uptake*.” |
| 3. | Please include 2 or 3 figures at least to represent your results in the revised manuscript. | Two figures were included in the manuscript:  Figure 1 – PRISMA flowchart showing the study selection  Figure 2 – Single-arm meta-analysis showing the outcome of the present review |
