# Supplementary material for: COVID-19 vaccination acceptance among dental students and dental practitioners: A systematic review and meta-analysis
Source: PLoS One. 2022 Apr 19;17(4):e0267354. doi: 10.1371/journal.pone.0267354 (PMC9017896; doi:10.1371/journal.pone.0267354)
Supplement: Attachment — Submitted filename: Response to Reviewers.docx [file pone.0267354.s006.docx]

**Response to Reviewers**

The authors would like to thank the editor and reviewers for considering the manuscript and their valuable comments. The amendments are made in the revised manuscript.

| **No.** | **Reviewer comment** | **correction** |
| --- | --- | --- |
| Editor | | |
| 1. | Please review your reference list to ensure that it is complete and correct. If you have cited papers that have been retracted, please include the rationale for doing so in the manuscript text or remove these references and replace them with relevant current references. Any changes to the reference list should be mentioned in the rebuttal letter that accompanies your revised manuscript. If you need to cite a retracted article, indicate the article’s retracted status in the References list and also include a citation and full reference for the retraction notice. | The reference list was amended accordingly. |
| Reviewer 3 | | |
| 1. | **Abstract:**  With 334 words, this section is much too long. Please stick to the Journal guidelines and reduce to the word maximum which is 300.  - With your revision, give priority to the results.  - With your Conclusions, please stick exclusively to your revised aims. Do not simply repeat your results here. Instead, provide a reasonable and generalizable extension of your outcome. | Dear reviewer, the authors attempted to reduce the number of words, and we agreed to use the full name, such as Joanna Briggs Institute, rather than JBI. Hence, the word count may slightly exceed.  The authors did not repeat the results, but we provide a generalised outcome:  “*Despite the high degree of acceptance of COVID-19 vaccination among dental practitioners, dental students still demonstrated a poor acceptance*”  An extension of the outcome was also provided:  “*These findings highlighted that evidence-based planning with effective approaches is warranted to enhance the knowledge and eradicate vaccination hesitancy, particularly among dental students*” |
| 2. | **Intro**  - Would seem sound.  - Please add some thoughts on your rationale. Dentists/dental students are considered a part of the society. Consequently, why should they differ from the society? Please clarify your idea when starting this project. | The authors have addressed the reason in conducting such a study because dental practitioners and dental students are classified as high-risk group.  “*Dental practitioners are among the healthcare workers classified as high-risk of infection during the COVID-19 pandemic due to the nature of their profession and the close proximity of the dental team to the patients*”  Therefore, they should be distinguished from the general public or society in order to determine the real impact of COVID-19 vaccination acceptance among them. |
| 3. | **Results**  - "Finally, only 10 articles are included (...)" must read "Finally, only 10 articles were included (...)".  - Level of evidence of included studies would seem poor. Please comment in this aspect. Why did you include such poor papers? And why have those poor papers been published? Please discuss thoroughly.  - Again, please stick to Journal guidelines. "(P=0.006)" must read "(p = 0.006)". "p" must be italicized.  - "No significant differences were found for both dental students (P=0.006) (...)." "(p = 0.006)" would indicate a significant difference, don't you think so? | The word ‘are’ had been changed to ‘were’.  The authors would like to thank the reviewer for raising this concern. Due to the nature of the study design, the level of evidence of the included cross-sectional studies appeared to be poor, as none of the research involved blinding the investigators or assessors.  Nonetheless, the included cross-sectional studies are still valuable as they can aid in determining the degree of acceptance of COVID-19 vaccination during this specific period.  The author also added the phrase:  “…*due to a lack of blinding among the investigators or assessors*”  Please accept the authors' sincere apologies for the 'p = 0.06' error. |
| 5. | - "To the best of the authors’ knowledge, it is also the first of its kind to critically summarise, analyse, and provide reliable evidence-based findings from existing research on the acceptability of COVID-19 vaccination in the dental community." Don't be too proud of your work, and do not re-repeat your pride. Compare to your Intro section ("To the best of the authors’ knowledge, no systematic review has been reported (...)"), and revise carefully. All readers will acknowledge your work, even if you will not show "the best of your knowledge"... | The authors have deleted the sentence at the first paragraph of the discussion section. |
| 6. | "(...) dental practitioners showed a high acceptance rate towards COVID-19 vaccination (81.1%) which is higher than the values reported in previous systematic reviews conducted on healthcare workers that ranged from 51% to 73% [8, 29]." I do not agree that this would be a "high acceptance rate". First, dental practitioners are a part of the medical science community, and this would call for a considerably higher rate. Second, there are countries with much higher rates, even with the normal population (see, for example, Arabic Emirates, Chile, or Portugal). Consequently, there is no need to be satisfied. Please discuss. | The authors would like to express their gratitude to the reviewer for sharing their thoughts on this matter. However, the authors believe that different religions and beliefs about COVID-19 immunisation do exist, as detailed in the introduction section. It is also unfair to assume that all dental practitioners across the world, although being part of the medical community, would have a high acceptance rate.  The authors were not particularly proud with the 81.8% acceptance rate; rather, we were comparing the acceptance rate to data reported among healthcare personnel in order to provide a more comprehensive picture to the readers. |
| 7. | "(...) a comprehensive literature search was performed in eight electronic databases to ensure that no relevant articles were missed." Please note that there are more databases. What about other languages? Please provide a sound assessment of the probability on how many papers have been missed, and how this could influence your outcome. | The authors did not impose any language restriction and the limitations were addressed in the manuscript. |
| 8. | Therefore, drawing solid conclusions on the global acceptance of the COVID-19 vaccine is impractical (...)." First, this would mean that publishing your paper will not be advocated. Second, why did you include a Conclusion section ("it can be concluded that dental practitioners, (...).") here? | The authors intended to provide recommendations for future research, advocating that more comparable studies be undertaken in order to reach a solid conclusion.  Of course, this does not imply that the existing manuscript has no value or merit. The authors believe that by publishing the current data, it would raise the awareness among dental researchers throughout the globe to delve deeper into this context.  Furthermore, conducting a systematic review allows readers to recognise the existing limitations.  The conclusion section was eliminated, and the points were relocated to the last paragraph of the discussion section. |
| 9 | Again, see comments given above, and revise carefully.  - Phrases given with "Furthermore, scientific research on the impact of (...)" to "(...) it is also essential to keep records of how dental students and dental practitioners respond to vaccinations and adjust vaccination strategies as required." would seem right, but are not considered Conclusions and must be copied & pasted to the Disc section. | The conclusion section was eliminated, and the points were relocated to the last paragraph of the discussion section. |
