# Supplementary material for: COVID-19 vaccination acceptance among dental students and dental practitioners: A systematic review and meta-analysis
Source: PLoS One. 2022 Apr 19;17(4):e0267354. doi: 10.1371/journal.pone.0267354 (PMC9017896; doi:10.1371/journal.pone.0267354)
Supplement: Attachment — Submitted filename: Response to Reviewers.docx [file pone.0267354.s007.docx]

**Response to Reviewers**

The authors would like to thank the editor and reviewers for considering the manuscript and their valuable comments. The amendments are made in the revised manuscript.

| **No.** | **Reviewer comment** | **correction** |
| --- | --- | --- |
| Editor | | |
| 1. | Please review your reference list to ensure that it is complete and correct. If you have cited papers that have been retracted, please include the rationale for doing so in the manuscript text or remove these references and replace them with relevant current references. Any changes to the reference list should be mentioned in the rebuttal letter that accompanies your revised manuscript. If you need to cite a retracted article, indicate the article’s retracted status in the References list and also include a citation and full reference for the retraction notice | The reference list was amended accordingly.  The second reference (No.2) has been changed. |
| Reviewer 1 | | |
| 1. | In the study selection section, the authors mention one of the exclusion criteria as " poor data reported on acceptance or hesitancy level." Please report how authors have differentiated between poor data and sound data. Also, what parameters are considered to determine this selection? | The authors have amended the following sentence for better understanding:  “*Mean and standard deviation on the acceptance or hesitancy level are not reported*” |
| 2. | The English of the manuscript may be improved. There are instances in the manuscript where the construction of sentences may be better (Minor). | The manuscript has been proofread by a native English speaker. |
| 3. | The studies considered for the study are from different countries. The number of COVID-19 cases (danger) and the availability of the vaccine to citizens are different in each country. Please discuss these factors in the discussion. | The authors appreciate the suggestion raised by the reviewer and have included some points in the discussion:  “*Furthermore, despite growing evidence of the safety and effectiveness of presently used vaccines …… continue to rise as vaccine availability increases [37].”* |
| Reviewer 3 | | |
| 1. | Maximum word count is 300. Again, please shorten, and note that this is due to the various databases and must be revised. | The authors have revised the abstract to 300 words. |
